# Top 100 Cited Publications in the Field of Temporomandibular Disorders: A Bibliometric Analysis

**DOI:** 10.3389/froh.2022.864519

**Published:** 2022-04-27

**Authors:** Yaser Al-Sharaee, Essam Ahmed Al-Moraissi, Nikolaos Christidis, Endi Lanza Galvão, Saulo Gabriel Moreira Falci

**Affiliations:** ^1^Department of Oral and Maxillofacial Surgery, Faculty of Dentistry, Thamar University, Dhamar, Yemen; ^2^Division of Oral Diagnostics and Rehabilitation, Department of Dental Medicine, Karolinska Institutet, Huddinge, Sweden; ^3^Scandinavian Center for Orofacial Neurosciences, Huddinge, Sweden; ^4^Oral and Maxillofacial Section, Department of Dentistry, Universidade Federal dos Vales Do Jequitinhonha e Mucuri, São Paulo, Brazil

**Keywords:** temporomandibular disorders, citation, bibliometric analyses, etiology, temporomandibular joint

## Abstract

**Background:**

The aim of this bibliometric research was to identify and analyze the top 100 cited publications in the field of temporomandibular disorders (TMD) in order to guide any professional level with interest in this topic by mapping the current trends in the field of TMD.

**Materials and Methods:**

The Clarivate Analytics' Web of Science database was used to find the top 100 most cited papers in the field of TMD, published from the year 2000 to November 18, 2021, with MeSH terms in the search strategy. Data extracted were ranking, title, main author, institution, publication year, a total of citations, citation average per year, the journal the study was published, journal impact factor, and the number of studies that each journal published. Further, also the percentage of the different study designs, the number of studies regarding a specific area within the field of TMD, and the number of studies per country were also calculated. A ranking of authors was also performed.

**Results:**

The top cited paper was a study on diagnostic criteria for TMD, with 1,287 citations published in 2014 in the Journal of Oral and Facial Pain and Headache which also had most of the top 100 cited publications. Eighty-one percent of the most cited studies were from the USA and Europe and 33% of the included studies were review articles.

**Conclusion:**

Taken together, since all papers were considered classic, one can draw the conclusion that researchers in 2000 onward in the field of TMD are interested in (a) diagnostic criteria, (b) TMD symptoms and mainly pain-related symptoms, (c) etiology and risk factors of TMD and mainly bruxism, and (d) treatment of TMD. However, topics such as imaging, occlusion, tissue engineering, and disk displacements are presently not as popular.

## Introduction

There is a wish among scientists in the field of dental medicine, as in all other fields, to reach out with their research findings to other scientists, practitioners, students, to the community, and to decision-makers due to the hope that their findings can be used to increase the knowledge and understanding about conditions and treatments, to be part of the education, to affect decisions in the clinic, and also to guide the decision-makers [1].

In order to investigate if the publications do reach out, if they have any impact in the research field of interest, or even if they affect decision making [2], one has to conduct scientometrics. Scientometrics, or bibliometrics as it is called in the field of science, is commonly used since the 1960s to show what impact publications have [3]. One type of bibliometrics is citation analysis, which is an analysis that quantifies how many times a publication has been cited after its publication. One can say that the more cited a publication is, the greater impact it has in its specific field [4]. Hence, citation analysis can be an efficient tool to use to evaluate what impact a publication has in a specific field and therefore how important this publication is in that specific field [4]. However, one has to be cautious since there are indications that errors or misinterpretations from one publication can become a cited “truth” that can be transferred and continued with repeated citations, which in turn can negatively influence practice and policy [1]. Finally, one has also to consider that citation analysis only can be used to assess the impact the specific publication has on its field by quantifying the recognition, importance, and popularity of the topic, but it cannot show any indication of the quality of the content in the specific publication [5, 6].

There are many pain related and jaw (dys)functional conditions that can affect the orofacial region. The orofacial region is one of the most frequent locations for chronic pain conditions, with a prevalence of 7–11% [7]. These conditions are embraced under the umbrella term temporomandibular disorders (TMD) which include not just chronic pain in conditions in the orofacial region affecting the temporomandibular joint (TMJ), the masticatory muscles (myalgia), and their associated structures, but also jaw functional limitations and occlusal aspects [8]. Painful TMDs are associated with restricted mouth opening capacity, pain upon chewing, muscle and joint soreness, and headache, i.e., impaired chewing ability [7]. Further, pain is a subjective individual experience that includes sensory, cognitive, emotional, and social dimensions [9], which, in other words, means that painful TMDs do not only lead to an unpleasant sensory experience, but is also accompanied by an unpleasant emotional experience with feelings of failure, misery, guilt, alienation, and even depression, i.e., a decreased quality of life [10].

In the field of TMD, there was a very important publication during the year 2014 that resulted in the worldwide accepted and used new diagnostic criteria for TMD for both clinicians and research [7]. The previous ones were from 1992, and they were mainly used for research in the field of TMD [11]. Although TMD is a broad field of dental medicine with a large amount of conducted research, there is still limited knowledge when it comes to etiology, pathophysiology, sex, age, and/or tissue differences, and treatment approaches to the various TMD conditions ([12]). Hence, it is of great interest not just for scientists, but also for clinicians and decision-makers to map the current trends in the field of TMD. To our knowledge, there are no studies that have performed such an analysis. Therefore, this bibliometric research aimed to identify and analyze the top 100 cited publications in the field of TMD to guide any professional level with interest in this topic by mapping the current trends in the field of TMD.

## Materials and Methods

A bibliometric analysis was performed to rank the top 100 cited papers related to the field of TMD. This study followed the same methodology applied in previous studies that analyzed the top 100 cited papers in robotic [13] and oral and maxillofacial surgery [14]. The search strategy was performed on November 18, 2021. The Clarivate Analytics' Web of Science database was used to find the top 100 most cited papers, and the following MeSH terms were used in the search strategy: “Disorder, Temporomandibular Joint,” “Disorders, Temporomandibular Joint,” “Joint Disorder, Temporomandibular,” “Joint Disorders, Temporomandibular,” “Temporomandibular Joint Disorder,” “TMJ Disorders,” “Disorder, TMJ,” “Disorders, TMJ,” “TMJ Disorder,” “Temporomandibular Disorders,” “Disorder, Temporomandibular,” “Disorders, Temporomandibular,” “Temporomandibular Disorder.”

In this bibliometric analysis, studies published from 2000 to the date of data extraction and the following study types were included: literature reviews, systematic reviews, cross-sectional cohort and case-control studies, randomized clinical trials, diagnostic accuracy studies, comparative studies, laboratory studies, technical report, genetic studies, methodological studies, questionnaire development, and animal studies.

However, some study types, namely, case reports, letters to the Editor, and papers not related to TMD, were excluded. Further, studies that were impossible to retrieve in full were also excluded. There was no restriction in language or journal of publication. The papers were sequentially screened from the most cited until a complete number of 100 papers were included. The screening process included reading titles, abstracts, and full text articles.

After screening each of the top 100 cited studies, the following data were collected: (1) ranking; (2) title; (3) the main author; (4) institution; (5) publication year; (6) total of citations; and (7) citation average per year. Further, (8) the name of the journals; (9) the journal impact factors, and (10) the number of studies that each journal published were also extracted. From the top 100 cited studies included, (11) the percentage of the different study designs; (12) the number of studies regarding a specific area within the field of TMD, and (13) the number of studies per country was also calculated. A ranking of authors was also performed. The authors who have published more than two studies were identified. Their main institution, country, number of studies published among the top 100 cited, and their total of citations were extracted.

Graphs were performed through Microsoft excel 2003. The field of investigation, study design, and the number of studies per country were graphed.

## Results

The initial search identified 8,927 publications. The 100 top-cited studies related to TMD are listed by rank order based on the number of citations in Table 1. The top-cited study was a review article related to diagnosis published in 2014 in the Journal of Oral and Facial Pain and Headache [7]. This study was finalized at the University of Minnesota (USA) and had 1,287 citations with the highest average citation per year (160.88 citations/year). The oldest study included was published in 2000, while the youngest in 2018. The top-cited author was “Schiffman EC” from the University of Minnesota, and this author published a total of three studies among the top 100 cited and had a total of 1,560 citations (Table 2).

**Table 1:** The top 100 cited studies in the field of temporomandibular disorders from year 2000 to 2021.

**Rank**	**Title**	**Main author**	**Institution**	**Year**	**Citations**	
					**Total**	**Average**
1	Diagnostic Criteria for Temporomandibular Disorders (DC/TMD) for Clinical and Research Applications: Recommendations of the International RDC/TMD Consortium Network and Orofacial Pain Special Interest Group	E. Schiffman	University of Minnesota	2014	1,287	160.88
2	Overlapping conditions among patients with chronic fatigue syndrome, fibromyalgia, and temporomandibular disorder	L. A. Aaron	University of Washington	2000	383	17.41
3	Bruxism physiology and pathology: an overview for clinicians	G. J. Lavigne	Université de Montréal	2008	380	27.14
4	Degenerative disorders of the temporomandibular joint: etiology, diagnosis, and treatment	E. Tanaka	University of Tokushima	2008	374	26.71
5	Mediators, moderators, and predictors of therapeutic change in cognitive-behavioral therapy for chronic pain	J. A. Turner	University of Washington	2007	330	22
6	Research diagnostic criteria for temporomandibular disorders (RDC/TMD): development of image analysis criteria and examiner reliability for image analysis	A. Mansur	University of Minnesota	2009	325	25
7	Research diagnostic criteria for temporomandibular disorders: a systematic review of axis I epidemiologic findings	D. Manfredini	University of Padova	2011	305	27.73
8	Medical Progress: Temporomandibular Disorders	S. J. Scrivani	Massachusetts General Hospital	2008	299	21.36
9	Bruxism is mainly regulated centrally, not peripherally	F. Lobbezoo	Academic Center for Dentistry Amsterdam (ACTA)	2001	280	13.33
10	Changes in temporomandibular pain and other symptoms across the menstrual cycle	L. LeResche	University of Washington	2003	260	13.68
11	Idiopathic pain disorders - Pathways of vulnerability	L. Diatchenko	University of North Carolina	2006	259	16.19
12	International consensus on the assessment of bruxism: Report of a work in progress	F. Lobbezoo	Academic Center for Dentistry Amsterdam (ACTA)	2018	254	63.5
13	Review of aetiological concepts of temporomandibular pain disorders: toward a biopsychosocial model for integration of physical disorder factors with psychological and psychosocial illness impact factors	T. I. Suvinen	University of Helsinki	2005	236	13.88
14	Role of Psychosocial Factors in the Etiology of Bruxism	D. Manfredini	University of Padova	2009	233	17.92
15	Risk factors for diagnostic subgroups of painful temporomandibular disorders (TMD)	G. J. Huang	University of Washington	2002	233	11.65
16	Relationship between bruxism and temporomandibular disorders: a systematic review of literature from 1998 to 2008	D. Manfredini	University of Padova	2010	222	18.5
17	Craniofacial muscle pain: Review of mechanisms and clinical manifestations	P. Svensson	Aalborg University	2001	222	10.57
18	Epidemiology of Bruxism in Adults: A Systematic Review of the Literature	D. Manfredini	University of Padova	2013	218	24.22
19	A longitudinal epidemiologic study of signs and symptoms of temporomandibular disorders from 15 to 35 years of age	T. Magnusson	Jönköping University	2000	212	9.64
20	Prevalence of temporomandibular disorder subtypes, psychologic distress, and psychosocial dysfunction in Asian patients	A. U. J. Yap	University of Singapore	2003	211	11.11
21	Enhanced Medial Prefrontal-Default Mode Network Functional Connectivity in Chronic Pain and Its Association with Pain Rumination	A. Kucyi	University of Toronto	2014	210	26.25
22	Deficiency in endogenous modulation of prolonged heat pain in patients with Irritable Bowel Syndrome and Temporomandibular Disorder	C. D. King	University of Florida	2009	206	15.85
23	Painful Temporomandibular Disorder: Decade of Discovery from OPPERA Studies	G. D. Slade	University of North Carolina	2016	185	30.83
24	Psychological Factors Associated With Development of TMD: The OPPERA Prospective Cohort Study	R. B. Fillingim	University of Florida	2013	184	20.44
25	Management of TMD: evidence from systematic reviews and meta-analyses	T. List	Malmö University	2010	183	15.25
26	Oro-facial pain in the community: prevalence and associated impact	T. V. Macfarlane	University of Manchester	2002	181	9.05
27	Radiographic examination of the temporomandibular joint using cone beam computed tomography	K. Tsiklakis	University of Athens	2004	179	9.94
28	Clinical Findings and Pain Symptoms as Potential Risk Factors for Chronic TMD: Descriptive Data and Empirically Identified Domains from the OPPERA Case-Control Study	R. Ohrbach	University at Buffalo	2011	178	16.18
29	Prevalence of temporomandibular dysfunction and its association with Malocclusion in children and adolescents: An epidemiologic study related to specified stages of dental development	B. Thilander	Göteborg University	2002	178	8.9
30	Short- and long-term efficacy of brief cognitive-behavioral therapy for patients with chronic temporomandibular disorder pain: A randomized, controlled trial	J. Á. Turner	University of Washington	2006	176	11
31	Relationship of pain and symptoms to pubertal development in adolescents	L. LeResche	University of Washington	2005	172	10.12
32	High prevalence of temporomandibular joint arthritis at disease onset in children with juvenile idiopathic arthritis, as detected by magnetic resonance imaging but not by ultrasound	P. F. Weiss	University of Pennsylvania	2008	170	12.14
33	A systematic review of the effectiveness of physical therapy interventions for temporomandibular disorders	M. L. McNeely	University of Alberta	2006	170	10.63
34	A Randomized clinical trial using Research Diagnostic Criteria for Temporomandibular Disorders-Axis II to target clinic cases for a tailored self-care TMD treatment program	S. F. Dworkin	University of Washington	2002	163	8.15
35	Temporomandibular disorders and oral health-related quality of life. A systematic review	L. Dahlstrom	Göteborg University	2010	162	13.5
36	Temporomandibular disorders and hormones in women	M. P. Warren	Columbia University	2001	161	7.67
37	Current Understanding of Pathogenesis and Treatment of TMJ Osteoarthritis	X. D. Wang	Peking University	2015	156	22.29
38	A prospective investigation over two decades on signs and symptoms of temporomandibular disorders and associated variables. A final summary	T. Magnusson	Jönköping University	2005	155	9.12
39	Sleep Disorders and their Association with Laboratory Pain Sensitivity in Temporomandibular Joint Disorder	M. T. Smith	Johns Hopkins University	2009	154	11.85
40	Reliability, validity, and clinical utility of the Research Diagnostic Criteria for Temporomandibular Disorders Axis II scales: Depression, non-specific physical symptoms, and graded chronic pain	S. F. Dworkin	University of Washington	2002	153	7.65
41	Group differences in pain modulation: pain-free women compared to pain-free men and to women with TMD	E. E. Bragdon	University of North Carolina	2002	151	7.55
42	Assessment of bruxism in the clinic	K. Koyano	Kyushu University	2008	150	10.71
43	Different Pain, Different Brain: Thalamic Anatomy in Neuropathic and Non-Neuropathic Chronic Pain Syndromes	S. M. Gustin	University of Sydney	2011	148	13.45
44	Early diagnosis of temporomandibular joint involvement in juvenile idiopathic arthritis: a pilot study comparing clinical examination and ultrasound to magnetic resonance imaging	L. Mueller	University of Zürich	2009	148	11.38
45	A randomized clinical trial of a tailored comprehensive care treatment program for temporomandibular disorders	S. F. Dworkin	University of Washington	2002	148	7.4
46	Pathophysiology of TMD pain - basic mechanisms and their implications for pharmacotherapy	B. E. Cairns	University of British Columbia	2010	147	12.25
47	The etiology of temporomandibular disorders: Implications for treatment	C. S. Greene	University of Illinois	2001	146	6.95
48	A systematic review of the effectiveness of exercise, manual therapy, electrotherapy, relaxation training, and biofeedback in the management of temporomandibular disorder	M. S. Medlicott	University of British Columbia	2006	145	9.06
49	Expanding the taxonomy of the diagnostic criteria for temporomandibular disorders	C. C. Peck	University of Sydney	2014	143	17.88
50	Temporomandibular disorders in relation to craniofacial dimensions, head posture and bite force in children selected for orthodontic treatment	L. Sonnesen	University of Copenhagen	2001	143	6.81
51	Reliability of clinical temporomandibular disorder diagnoses	M. T. John	University of Leipzig	2005	142	8.35
52	Potential Psychosocial Risk Factors for Chronic TMD: Descriptive Data and Empirically Identified Domains from the OPPERA Case-Control Study	R. B. Fillingim	University of Florida	2011	141	12.82
53	The Research Diagnostic Criteria for Temporomandibular Disorders. V: Methods Used to Establish and Validate Revised Axis I Diagnostic Algorithms	E. L. Schiffman	University of Minnesota	2010	141	11.75
54	Influence of psychological factors on risk of temporomandibular disorders	G. D. Slade	University of Adelaide	2007	141	9.4
55	Three major haplotypes of the beta 2 adrenergic receptor define psychological profile, blood pressure, and the risk for development of a common musculoskeletal pain disorder	L. Diatchenko	University of North Carolina	2006	141	8.81
56	Depression and somatization in patients with temporomandibular disorders	A. U. J. Yap	University of Singapore	2002	140	7
57	Orofacial Pain Prospective Evaluation and Risk Assessment Study - The OPPERA Study	W. Maixner	University of North Carolina	2011	135	12.27
58	Pain Sensitivity Risk Factors for Chronic TMD: Descriptive Data and Empirically Identified Domains from the OPPERA Case Control Study	J. D. Greenspan	University of Maryland	2011	133	12.09
59	The Research Diagnostic Criteria for Temporomandibular Disorders. I: Overview and Methodology for Assessment of Validity	E. L. Schiffman	University of Minnesota	2010	132	11
60	Are headache and temporomandibular disorders related? A blinded study	V. Ballegaard	University of Copenhagen	2008	131	9.36
61	Contributing factors to chronic myofascial pain: a case-control study	A. M. Velly	McGill University	2003	130	6.84
62	Temporomandibular joint disk displacement: Comparison in asymptomatic volunteers and patients	T. A. Larheim	University of Oslo	2001	130	6.19
63	Quantification and validation of predictive values of occlusal variables in temporomandibular disorders using a multifactorial analysis	A. G. Pullinger	University of California	2000	130	5.91
64	Chronic myofascial temporomandibular pain is associated with neural abnormalities in the trigeminal and limbic systems	J. W. Younger	University of California	2010	128	10.67
65	Bruxism: its multiple causes and its effects on dental implants - an updated review	F. Lobbezoo	Academic Center for Dentistry	2006	125	7.81
66	TMJ disorders: Future innovations in diagnostics and therapeutics	S. Wadhwa	University of Connecticut	2008	123	8.79
67	Symptoms of Temporomandibular Disorders in the Population: An Epidemiological Study	D. A. de Godoi Goncalves	University of São Paulo	2010	122	10.17
68	Drugs and bruxism: A critical review	E. Winocur	Tel Aviv University	2003	122	6.42
69	Oral health-related quality of life in patients with temporomandibular disorders	M. T. John	University of Leipzig	2007	120	8
70	Predictors of onset of facial pain and temporomandibular disorders in early adolescence	L. LeResche	University of Washington	2007	120	8
71	A 20-year follow-up of signs and symptoms of temporomandibular disorders and malocclusions in subjects with and without orthodontic treatment in childhood	I. Egermark	Göteborg University	2003	118	6.21
72	A 20-year longitudinal study of subjective symptoms of temporomandibular disorders from childhood to adulthood	I. Egermark	Göteborg University	2001	118	5.62
73	Analysis of stimulus-evoked pain in patients with myofascial temporomandibular pain disorders	P. Svensson	Aalborg University	2001	117	5.57
74	Depression, pain, exposure to stressful life events, and long-term outcomes in temporomandibular disorder patients	S. M. Auerbach	Virginia Commonwealth University	2001	116	5.52
75	Diagnosis and Treatment of Temporomandibular Disorders	R. L. Gauer	Womack Medical Center	2015	115	16.43
76	Oral parafunctions as risk factors for diagnostic TMD subgroups	A. Michelotti	University of Napoles	2010	115	9.58
77	Headache and Symptoms of Temporomandibular Disorder: An Epidemiological Study	D. A. G. Goncalves	University of São Paulo	2010	115	9.58
78	Bilateral Widespread Mechanical Pain Sensitivity in Women With Myofascial Temporomandibular Disorder: Evidence of Impairment in Central Nociceptive Processing	C. Fernandez-de-las-Penas	Universidad Rey Juan Carlos	2009	115	8.85
79	Development of a brief and effective temporomandibular disorder pain screening questionnaire Reliability and validity	Y. M. Gonzalez	University of Buffalo	2011	114	10.36
80	Viscoelastic characterization of the porcine temporomandibular joint disc under unconfined compression	K. D. Allen	Rice University	2006	114	7.13
81	The role of parafunctions, emotions and stress in predicting facial pain	A. G. Glaros	Kansas City University	2005	114	6.71
82	Potential Genetic Risk Factors for Chronic TMD: Genetic Associations from the OPPERA Case Control Study	Smith, Shad B	University of North Carolina	2011	112	10.18
83	The roles of beliefs, catastrophizing, and coping in the functioning of patients with temporomandibular disorders	Turner, JÁ	University of Washington	2001	112	5.33
84	Temporomandibular Disorders: A Review of Etiology, Clinical Management, and Tissue Engineering Strategies	Murphy, Meghan K	University of California	2013	111	12.33
85	Evidence for up-regulated central nociceptive processing in patients with masticatory myofascial pain	Sarlani, E	University of Maryland	2004	111	6.17
86	Longitudinal outcome of temporomandibular disorders: A 5-year epidemiologic study of muscle disorders defined by Research Diagnostic Criteria for Temporomandibular Disorders	Rammelsberg, P	University of Heidelberg	2003	111	5.84
87	Widespread pain as a risk factor for dysfunctional temporomandibular disorder pain	John, MT	Martin Luther University	2003	111	5.84
88	A comparison of human umbilical cord matrix stem cells and temporomandibular joint condylar Chondrocytes for tissue engineering temporomandibular joint condylar cartilage	Bailey, Mark M	University of Kansas	2007	110	7.33
89	Importance of proinflammatory cytokines in synovial fluid from 121 joints with temporomandibular disorders	K. Kaneyama	Kanazawa Medical University	2002	109	5.45
90	The relationship between headache and symptoms of temporomandibular disorder in the general population	R. Ciancaglini	Univeristy of Milan	2001	109	5.19
91	Adrenergic Dysregulation and Pain With and Without Acute Beta-Blockade in Women With Fibromyalgia and Temporomandibular Disorder	K. C. Light	University of Utah	2009	108	8.31
92	The efficacy of traditional, low-cost and nonsplint therapies for temporomandibular disorder: A randomized controlled trial	E. Truelove	University of Washington	2006	108	6.75
93	Efficacy of temporomandibular joint arthrocentesis with and without injection of sodium hyaluronate in treatment of internal derangements	G. H. Alpaslan	Gazi University	2001	108	5.14
94	Oral appliances in the management of temporomandibular disorders	G. D. Klasser	University of Illinois	2009	107	8.23
95	Temporomandibular Joint and Muscle Disorder-type Pain in US Adults: The National Health Interview Survey	U. Isong	University of California	2008	107	7.64
96	Temporomandibular joint arthritis in juvenile idiopathic arthritis: Prevalence, clinical and radiological signs, and relation to dentofacial morphology	A. D. Billiau	University of Leuven	2007	107	7.13
97	Counseling and physical therapy as treatment for myofascial pain of the masticatory system	A. De Laat	University of Leuven	2003	107	5.63
98	Systematic review of population-based epidemiological studies of oro-facial pain	T. V. Macfarlane	University of Manchester	2001	107	5.1
99	Need for occlusal therapy and prosthodontic treatment in the management of temporomandibular disorders. Part I. Occlusal interferences and occlusal adjustment	J. Á. De Boever	University of Gent	2000	107	4.86
100	Situational Versus Dispositional Measurement of Catastrophizing: Associations With Pain Responses in Multiple Samples	C. M. Campbell	Johns Hopkins University	2010	106	8.83

**Table 2:** The 15 authors with most citations among the top 100 cited studies in the field of temporomandibular disorders from year 2000 to 2021.

**Rank**	**First author**	**Institution**	**Country**	**Number of articles**	**Total of citations**
1	E. C. Schiffman	University of Minnesota	USA	3	1,560
2	D. Manfredini	University of Padova	Italy	4	978
3	F. Lobbezoo	Academic Center for Dentistry Amsterdam (ACTA)	The Netherlands	3	659
4	J. Á. Turner	University of Washington	USA	3	618
5	L. LeResche	University of Washington	USA	3	552
6	S. F. Dworkin	University of Washington	USA	3	464
7	L. Diatchenko	University of North Carolina	USA	2	400
8	M. T. John	University of Leipzig	Germany	3	373
9	T. Magnusson	Jönköping University	Sweden	2	367
10	G. D. Slade	University of Adelaide	Australia	2	326
11	R. B. Fillingim	University of Florida	USA	2	325
12	A. U. J. Yap	University of Singapore	Singapore	2	351
13	P. Svensson	Aalborg University	Denmark	2	339
14	T. V. Macfarlane	University of Manchester	England	2	288
15	I. Egermark	Göteborg University	Sweden	2	236

**Figure 1:**
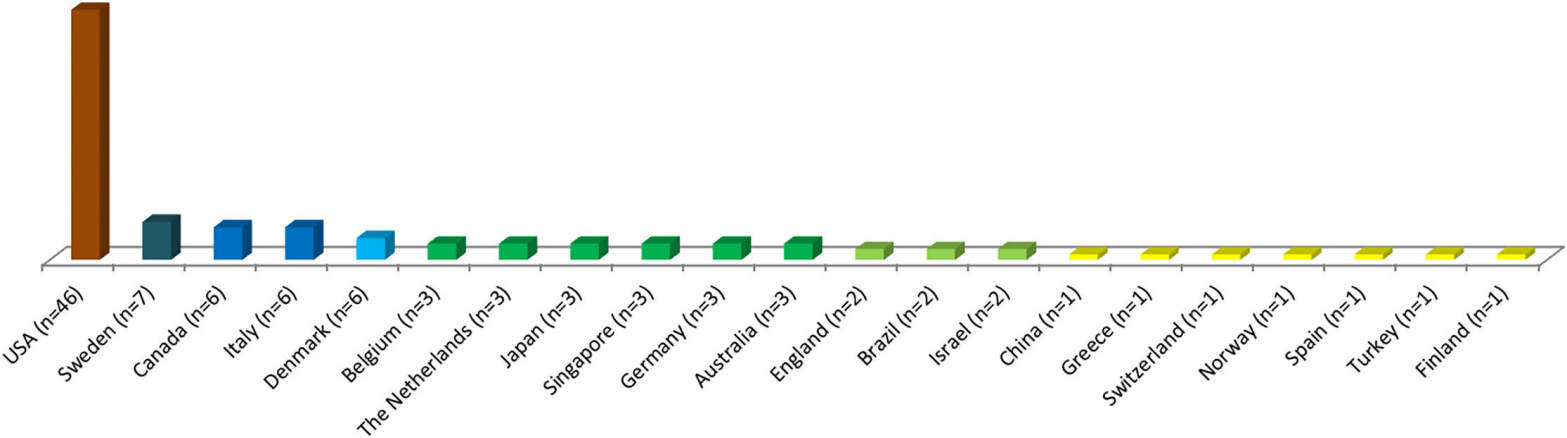
Number of top 100 cited studies in the field of temporomandibular disorders published per country from 2000 to 2021.

A total of 17,434 citations were found among the included studies. Of the 100 top-cited, 46 were published by researchers from the USA, followed by Sweden with a total of 7 published studies. Fifty-four publications were from America, 36 from Europe, 7 from Asia, and 3 from Oceania (Figure 1).

A total of 36 journals were found among the 100 top-cited papers. The Journal of Orofacial Pain had 18 published studies among the top 100 cited, or, in fact, 19 since it changed its name to Journal of Oral and Facial Pain and Headache in 2014. The highest impact factor found was from one publication in the journal New England Journal of Medicine (91.253) followed by the journal Archives of Internal Medicine (17.333) (Table 3).

**Table 3:** Ranking of journal based on number of publications.

**Rank**	**Journal**	**Impact factor**	**Number of papers published**
1	Journal of orofacial pain	2.824	18
2	Pain	6.961	14
3	Journal of oral rehabilitation	3.837	10
4	Journal of pain	5.828	9
5	Journal of dental research	6.116	5
6	Oral surgery oral medicine oral pathology oral radiology and endodontology	1.457	4
7	Acta odontologica scandinavica	2.331	3
8	Journal of the American Dental Association	3.634	3
9	Journal of neuroscience	6.167	2
10	Angle orthodontist	2.079	2
11	Physical therapy	3.140	2
12	Journal of prosthetic dentistry	3.426	2
13	Journal of oral and maxillofacial surgery	1.895	2
14	Journal of dentistry	4.379	2
15	Journal of oral and facial pain and headache	1.871	1
16	Archives of internal medicine	17.333	1
17	New England journal of medicine	91.253	1
18	European journal of pain	3.934	1
19	Community dentistry and oral epidemiology	3.383	1
20	Dentomaxillofacial radiology	2.419	1
21	Arthritis and rheumatism	7.379	1
22	Cells tissues organs	2.481	1
23	Sleep	5.849	1
24	Rheumatology	7.580	1
25	European journal of orthodontics	3.934	1
26	American journal of medical genetics part b-neuropsychiatric genetics	3.568	1
27	Cephalalgia	6.295	1
28	Radiology	11.105	1
29	Journal of dental education	2.264	1
30	American family physician	3.292	1
31	Headache	5.887	1
32	Journal of biomechanics	2.712	1
33	International journal of oral and maxillofacial implants	2.804	1
34	Tissue engineering	3.508	1
35	British journal of oral and maxillofacial surgery	1.651	1
36	Journal of rheumatology	4.666	1

**Figure 2:**
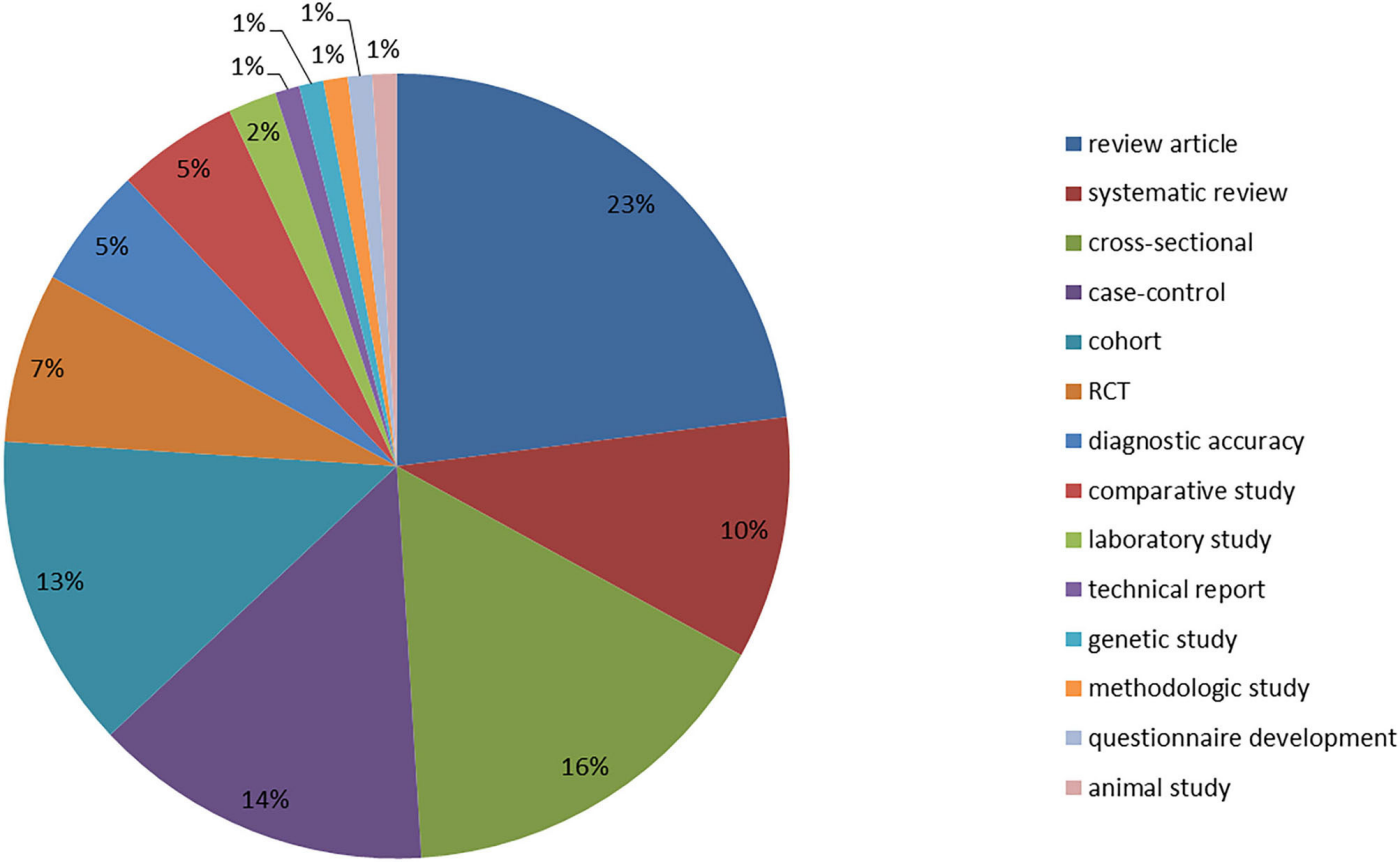
Percentage distribution of study types. Some of the studies could not be referred to a specific study type, thus they were clustered based on the content, e.g., diagnostic accuracy, questionnaire development, and review article (non-systematic reviews of any kind). RCT, randomized controlled trial.

Thirty-three percent of the studies included were review papers, 43% were observational studies, and 12% were clinical studies (Figure 2).

**Figure 3:**
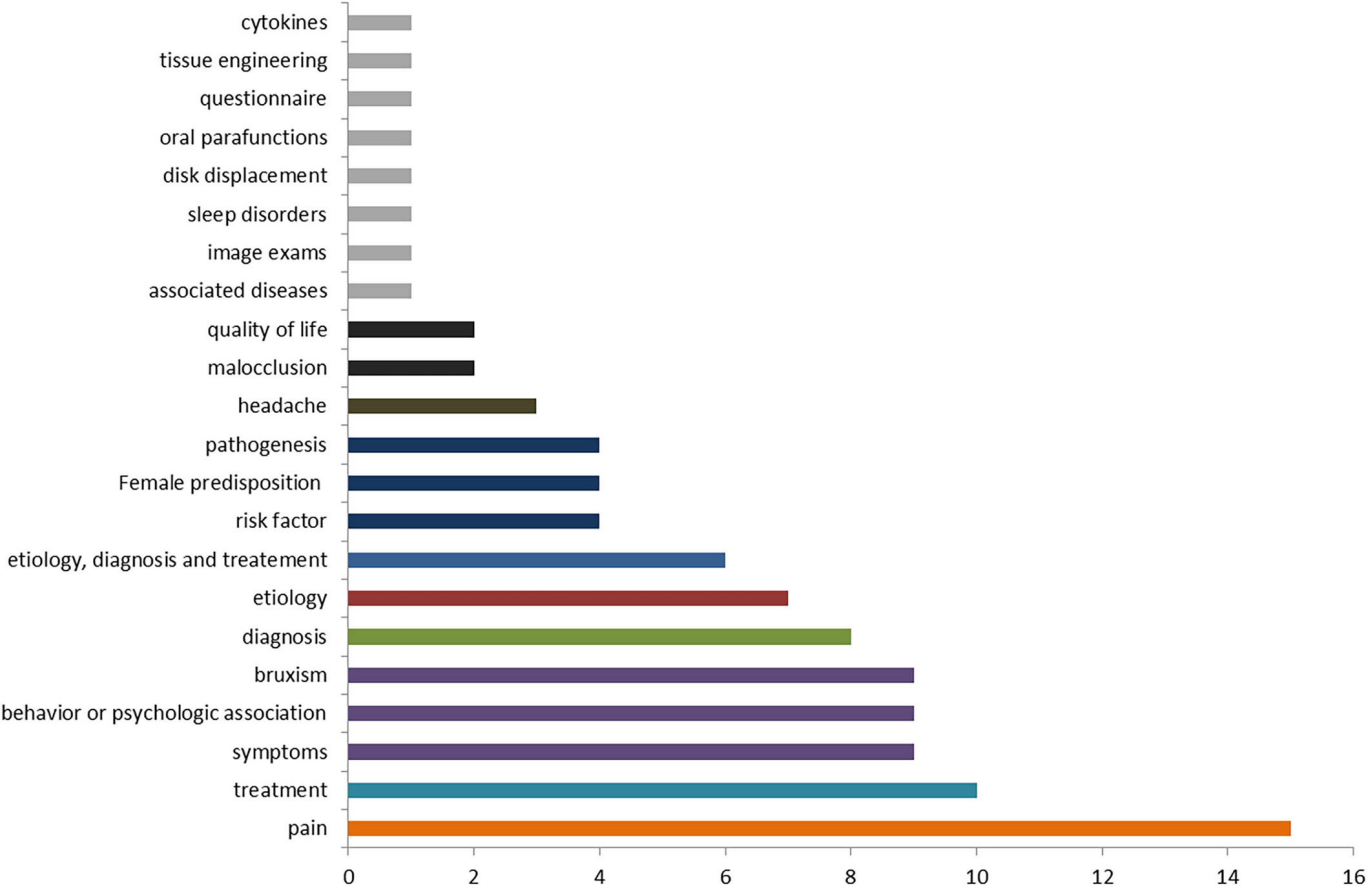
Topics within the field of temporomandibular disorders covered in the 100 top-cited studies.

When it comes to the field of investigation, i.e., which topic within the field of TMD, the most common topic was “pain” with a total of 15 publications, followed by the topic “treatment” and bruxism with a total of 10 publications (Figure 3). When it comes to bruxism, six out of the 18 cited studies concerned that topic.

## Discussion

In this bibliometric analysis, the top cited paper in the field of TMD the last 20 years, i.e., from the millennial shift (the year 2000), is an American publication setting new and improved diagnostic criteria for TMD (DC/TMD) [7]. This was not a surprising finding, since these diagnostic criteria are used worldwide by both clinicians and researchers in the field of TMD. Further, journals in the field of TMD, for instance, the Journal of Oral and Facial Pain and Headache (http://www.quintpub.com/journals/ofph/index.php), require researchers to adhere to the methodology, terminology, and diagnostic criteria as set by this top-cited study by Schiffman et al. [7].

As in the field of oral and maxillofacial surgery [14], most (81 of 100) of the top-cited studies were from the USA and Europe. However, this was not a surprising finding since it has been shown that most of the top-cited or top-ranked studies are conducted in countries with better economic rankings [15, 16]. This finding can also explain the fact that 13 of the 15 most cited authors are from the USA and Europe. Further, although the publications are spread between 36 journals, one journal has attracted as many as 20% of the top 100 cited studies. One explanation could be that it is the “Official Journal of the American Academy of Orofacial Pain, the European Academy of Orofacial Pain and Dysfunction, the Asian Academy of Craniomandibular Disorders, and the Australian and New Zealand Academy of Orofacial Pain” (http://www.quintpub.com/journals/ofph/index.php). Still, this is surprising since authors strive to publish their studies in journals with high impact factors since a high impact factor also is considered as an indication of a study with great impact and high quality [5, 6]. However, considering all 36 journals among the 100 top cited studies, this analysis shows that authors in the field of TMD tend to strive for journals with high impact factors and, when possible, also choose journals that have higher impact factors although not TMD specific, such as New England Journal of Medicine and Archives of Internal Medicine. This is possible due to the multifaceted character of TMD conditions and the multidisciplinary nature of the therapeutic approach to these conditions [7, 8, 10]. This is also consistent with the fact that researchers tend to cite studies from journals with higher impact factors, where the journal impact factor answers for 59% of the variation in the number of citations [17].

Researchers in all fields of science aim to communicate their findings to other scientists, clinicians, and decision-makers [1]. Using this citation analysis report to assess the impact the specific articles have on the field of TMD, its importance, and popularity, one can understand why the new diagnostic criteria (DC/TMD) by Schiffman et al. [7] is top cited. It is a study providing sensitive and specific protocols to examine and diagnose patients with TMD for undergraduate dental students and clinicians worldwide, for researchers for the possibility to compare results and outcomes from different studies in different countries, and for decision-makers to use for treatment guidelines. However, this report cannot reflect the quality of the content in the specific studies [5, 6].

It was not surprising that the majority of the top cited studies were published between the years of 2007 and 2011 since it has been shown that studies are cited just sparingly with few citations in the first years, followed by a peak of citations just before a study-age of 10 years [18]. Although the outcome of bibliometric analyses of this kind are criticized for being affected by the impact of time [19], this is not the case in this study since the bulk of most cited papers are from the years 2007 to 2018 and not from 2000 to 2006.

Another interesting finding was that all of the top 100 cited studies had 100 citations or more, and are thus classic studies [2]. Classic studies are considered to have a great impact [2], of which their outcomes can be used to affect decisions and guide the readers in their decision making [1]. One must not forget the possibility of self-citations to be a possible explanation to the high number of classical studies. However, previous studies have discussed this and came to the conclusion that there is no need for any revision of the journal citation metrics used in bibliometric analysis since the extent of self-citations were not related either to the number of co-authors or to the authors' productivity [20, 21].

Taken together, since all papers are considered classic ones, we can conclude that researchers in the year 2000 and onward in the field of TMD are interested in (a) diagnostic criteria, (b) TMD symptoms and mainly pain-related symptoms, (c) etiology and risk factors of TMD and mainly bruxism, and (d) treatment of TMD. However, topics such as imaging, occlusion, tissue engineering, and disk displacements are presently not as popular.

## Author Contributions

All authors contributed equally in this manuscript, thus participating in data collection, analysis, and interpretation, also authoring the introduction, methods, results, discussion, and read and approved the final version of the manuscript.

## Funding

This study was financed in part by the Coordenação de Aperfeiçoamento de Pessoal de Nível Superior (CAPES)—Brazil (Finance Code 001).

## Conflict of Interest

The authors declare that the research was conducted in the absence of any commercial or financial relationships that could be construed as a potential conflict of interest.

## Publisher's Note

All claims expressed in this article are solely those of the authors and do not necessarily represent those of their affiliated organizations, or those of the publisher, the editors and the reviewers. Any product that may be evaluated in this article, or claim that may be made by its manufacturer, is not guaranteed or endorsed by the publisher.
